# Master or Escape: Digitization-Oriented Job Demands and Crafting and Withdrawal of Chinese Public Sector Employees

**DOI:** 10.3390/bs15030378

**Published:** 2025-03-17

**Authors:** Huan Huang, Jiangyu Li

**Affiliations:** School of Public Administration, Sichuan University, Chengdu 610065, China; huanghuan@stu.scu.edu.cn

**Keywords:** digitization-oriented job demands, thriving at work, workplace anxiety, job crafting, work withdrawal, regulatory focus

## Abstract

Public sector employees face the profound impact of digital work demands, especially with the advancement of China’s digital government construction. This study explores the dual-edged consequence of digital job demands on the work behaviors of public sector employees in China by constructing a dual-path model. Structural equation modeling (SEM) was used to validate the data of 873 public sector employees. This study found that digital job demands increase civil servants’ thriving at work, facilitating their job-crafting behaviors and increasing their workplace anxiety, leading to their work withdrawal behavior. Furthermore, this study validates the moderating effects of promotion and preventive focus. This study provides managers in the public sector with valuable insights to develop digital job demands managing strategies and for civil servants to adapt their perceptions and behaviors in the digital context.

## 1. Introduction

In light of the growing prevalence of digital technology adoption, organizations are embracing technological digital innovations to enhance value (Chen et al., 2024). An organization’s digital transformation hinges on its members’ proficiency (Ghi et al., 2022). Employees must exhibit greater creative thinking and computer technical expertise in this setting (T.-J. Wu et al., 2025). In this context, the demands to master digital skills can be defined as digitization-oriented job demands. It has been demonstrated that when perceived as pressure, job demands can considerably influence employees’ psychological processes and behavioral responses (Hou & Fan, 2024). The rapid iteration and continuous application of digital technologies have resulted in a more direct and pronounced impact of digitization-oriented job demands on employees (P. Liu et al., 2024). Furthermore, it is critical to recognize that technology has social characteristics and that human workers are essential to its use and interaction (Y. Liu et al., 2024). Consequently, employee factors should be a primary consideration when confronted with digitization-oriented job demands (Parker & Grote, 2022).

In conclusion, there are two significant viewpoints of how organizations implementing digital technology affect their workforces. One asserts that prolonged exposure to technology may foster positive attitudes and behaviors (Hou & Fan, 2024), including enhanced employee agility (AlNuaimi et al., 2022), innovativeness (Riaz et al., 2018), and other beneficial outcomes (Ingusci et al., 2021). The other proposes that employees may also experience insecurity, incapacity, and stress (Ingusci et al., 2021). This can manifest as a reduction in job resilience (P. Liu et al., 2024). Furthermore, loneliness has been linked to an increase in negative behaviors such as off-duty insomnia and alcohol consumption (Tang et al., 2023). Both work withdrawal and job crafting can substantially impact organizational and employee growth (Bakker & de Vries, 2021; Rosse & Hulin, 1985). The work environment, the work nature itself, and the traits of the individual employee all affect the previously listed aspects (P. Liu et al., 2024). The extant literature suggests that changes in job characteristics and environment can encourage job-crafting behaviors (Leana et al., 2009) but could also result in the rise of disengagement behaviors (Zhang et al., 2023). These studies provide valuable references for our innovative research combining digitalization and employee job demands. The two voices, however, do not communicate well, and a unified vocabulary regarding the overall effects of digitization-oriented job demands on employees has not yet been established (P. Liu et al., 2024). Therefore, this study’s primary objective is to examine how digitization-oriented job demands affect employee behavior in two ways.

In China, due to the nature of government work and the construction of a digital government, public sector employees will probably be required to undertake a more significant proportion of digitization-oriented job demands (Jung & Camarena, 2024). On the one hand, the public sector employees’ core duties are typically more monotonous and repetitive (Chatterjee et al., 2023), and the positive impact of digital technology in addressing such job demands is more evident. Conversely, based on the vertical leadership structure of the Chinese public sector (P. Liu et al., 2024), the responsibility for the construction of digital government is distributed vertically to lower levels of government. Consequently, the public sector and its employees must incorporate greater digital effectiveness into their performance. This is accompanied by increased demand, a lack of flexibility in employees’ digital work, and a more pronounced negative impact of digitization-oriented job demands.

This study uses the Conservation of Resources (COR) Theory (T.-J. Wu et al., 2024) to explain how employee behavior is affected by digitization-oriented job demands. When individuals possess resources, they are motivated to protect current resources and acquire additional ones. Digitization-oriented job demands facilitate continuous learning of technologies and tools, incentivizing employees to acquire additional knowledge and skills (Y. Liu et al., 2024). In turn, employees have more resources to learn and grow, and are more likely to develop a sense of job thriving and engage in positive work behaviors (Chen et al., 2024). Conversely, it may also result in negative employee behavior aimed at protecting limited resources (Y. Liu et al., 2024). Some employees experience difficulties adapting to digitization-oriented job demands (Chatterjee et al., 2023). They may feel anxiety and depleted about the loss of resources and may perceive the demands as a threat. This may result in the display of defensive attitudes and behaviors (J. Wu et al., 2022). Considering the findings above, this study’s second goal was to explore how workplace anxiety and thriving at work mediate the relationship between employee behavior and digitization-oriented job demands.

If digitization-oriented job demands have a double-edged sword effect on employees’ psychology and behavior, when do the demands lead to resource enrichment and when do they lead to resource loss? According to the regulatory focus theory, individuals develop their own idiosyncratic regulatory focus as they grow up, and different regulatory focuses determine the differences in their emotions and attitudes when coping with stress and acquiring new resources (Lanaj et al., 2012). When confronted with the same task, individuals with a promotion regulatory focus experience greater emotional pleasure, seek progress and growth, and strive to alter the status quo to achieve success (P. Liu et al., 2024). These individuals are more concerned about access to resources and are more likely to evaluate digitization-oriented job demands as a challenge, increasing their psychological resources and generating positive states such as job prosperity. In addition, they are more likely to expect success, are eager to learn and acquire new resources, and are more likely to engage in job-crafting behaviors. On the other hand, people with a prevention focus are more focused on emotional safety, are afraid of bad things happening, and prefer to keep things the way they are to avert losses (Brenninkmeijer et al., 2010). They are more likely to perceive signals of loss of resources and tend to perceive digitization-oriented job demands as a threat, which depletes the individual’s psychological resources and generates negative emotions such as workplace anxiety, which may lead to avoidance behaviors (P. Liu et al., 2024; Zhao & Liu, 2024).Therefore, the current research’s third objective was to examine the possible dual impact of job expectations centered upon digitization on employee behavior, emphasizing the moderating role of regulatory focus.

**Figure 1:**
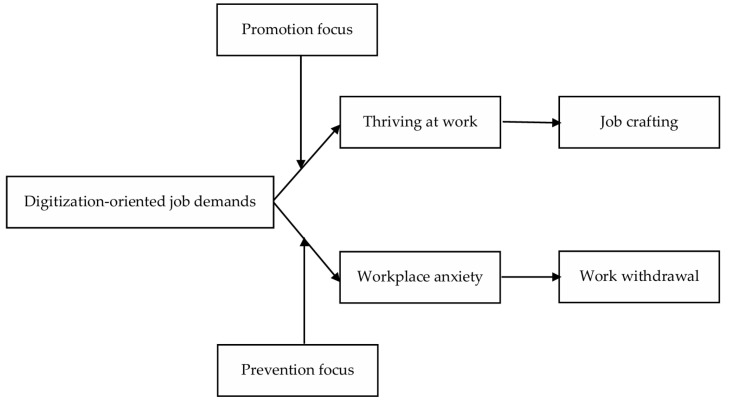
Research model diagram.

The current work significantly advances our understanding of theory and practice. Theoretically, this study creates a new dual-path framework for examining how employment expectations focused on digitalization affect employees’ psychology and behavior (as shown in Figure 1). From a pragmatic perspective, this study provides valuable suggestions to help managers and organizations better grasp the precise effects of job demands driven by digitalization on workers and improve management tactics for encouraging digital adoption.

## 2. Literature Review and Hypotheses Development

### 2.1. Job Crafting and Work Withdrawal Behaviors as a Result of Digitization-Oriented Job Demands

Job demands can be characterized as the necessity to update job skills and improve work competencies in light of social and organizational change (Li et al., 2023). Digitization-oriented job demands are defined as the need to possess specific digital skills (Hess et al., 2016) to adapt to digital changes within the organization’s structure, functions, processes, and mode of operation (Vial, 2019). Job crafting represents actions employees take to alter the physical task boundaries of a job, the cognitive task boundaries of a job, and the relational boundaries of a job (Demerouti et al., 2015). Integrating digital technologies into the workplace can be valuable for improving workflow management (Muneer et al., 2024). Existing research has shown that digital job demands in the public sector provide employees opportunities to acquire new technological tools, enabling them to view existing task processes and responsibilities more positively and proactively and adjust work methods (Ramakrishnan et al., 2024). For example, digital technologies facilitate convenient access to information for public servants, streamline the volume and content of repetitive and tedious tasks (Chatterjee et al., 2023). It also promotes the virtualization of certain workplaces, increases interactions between employees and AI systems, altering the way of life through which employees communicate with each other and their surroundings (Muneer et al., 2024). Therefore, this study suggests that in the Chinese public sector, digitization-oriented job demands will increase employees’ job-crafting behaviors.

Work withdrawal behaviors refer to actions taken by employees to keep a psychological or physical distance from their activities and work environment. Recent research has also demonstrated that organizational digital transformation may hinder proactive behaviors (Sandoval-Reyes et al., 2019). For example, employees who are unsure of the impact of digital-AI transformation may be hesitant to challenge the status quo and fear unintended consequences, so their proactive behavior such as taking charge is hindered (Pei et al., 2025). To navigate the skills and knowledge about technology (Ingusci et al., 2021), employees may experience stress, anxiety, uncertainty, and incapacity (Muneer et al., 2024). This can lead to disengaged behaviors like work withdrawal.

According to the COR theory, individuals maintain psychological stability and behavioral motivation by acquiring and protecting resources, which are the currency of behavior (Hobfoll, 2012). Individuals are more likely to invest in themselves and develop more resources when they have larger resources. On the other hand, they use defensive strategies to protect existing resources when faced with resource depletion (Hobfoll et al., 2018). In conjunction with COR theory, we argue that digitization-oriented job demands can create a resource–gain pathway that stimulates public sector employees’ job-crafting behaviors. First, employees are motivated to learn digital tools (e.g., AI assistants) (Khan et al., 2024), which reduce duplication of effort (Muneer et al., 2024), enabling employees to focus on high-value tasks, promoting innovative behavior (P. Liu et al., 2024). Second, digital technology allows employees to have higher time and space autonomy (e.g., flexible office), which increases their conditional resources (Uygungil-Erdogan et al., 2025). Such conditional resources reduce employees’ roles in conflict, which in turn stimulates organizational citizenship behavior (Hill et al., 2024). Finally, digitization-oriented job demands require employees to continue learning (e.g., participating in online training), which also contributes to employees’ abilities to acquire new resources, triggering a resource spiral effect, further enhancing employees’ mental stability and proactive behaviors.

Conversely, digitization-oriented job demands can also lead to public sector employees’ negative behaviors aimed at protecting limited resources (Y. Liu et al., 2024). Some employees may experience difficulty adapting promptly, resulting in cognitive loads that cumulatively trigger emotional exhaustion in the long term, prompting employees to reduce their efforts to conserve remaining resources (Demerouti & Bakker, 2023). The “always-on” nature of digital technologies may also lead to the crowding out of employees’ personal time by fragmented tasks (Khan et al., 2024), especially in the public sector where leaders are in a position of authority. This can lead to fewer opportunities for employee resource recovery. Finally, complex and changing technological demands may also trigger employees’ technological anxiety, further depleting their resources and ultimately inducing negative behaviors such as work withdrawal (J. Wu et al., 2022). This research proposes a two-way model (i.e., gain path and loss path) to clarify how digitization-oriented job demands impact employees’ work behaviors, considering the conclusions above. We put out the following theories considering the analysis above:

**H1a.**  *Digitization-oriented job demands is positively associated with job crafting*.

**H1b.**  *Digitization-oriented job demands is positively associated with work withdrawal*.

### 2.2. Resource Enrichment Pathways—The Mediating Role of Thriving at Work

Thriving at work refers to an individual’s level of learning (i.e., a gain in understanding and knowledge) and vitality (i.e., an increase in energy) within the framework of their job (Spreitzer et al., 2005). A sensation of usable energy and a perception of vigor (Nix et al., 1999) is known as vitality. Learning is a cognitive and affective state where knowledge and skills are acquired and applied (Elliott & Dweck, 1988). It is influenced by situational features (e.g., job stress) and agentic work behaviors (e.g., focus and exploration), so its conception does involve positive work behaviors. Based on existing research, we place more emphasis on the sense of job prosperity as a psychological state (Mansour & Tremblay, 2020), distinguishing it from the specific behavior of job crafting. Workers who lack vitality but are nevertheless dedicated to learning may feel worn out or exhausted. Those who are driven to work but do not have access to learning and development opportunities are more likely to stagnate (Demerouti & Bakker, 2023). Employees flourishing at work are more engaged, focused, and inclined to act better for the company (Yang et al., 2024). However, there is a paucity of research on how employee work thriving affects crafting behavior in the context of organizational digital transformation.

According to the COR theory, we put forth the proposition that digitization-oriented job demands may enhance employees’ thriving at work through gainful mechanisms. Related studies point out that work environments with high technological adaptability drive employees to reinvent their work by enhancing the sense of thriving at work (Kaltiainen & Hakanen, 2024). Firstly, digitization-oriented job demands help employees complete tasks more expediently and efficiently, thereby enhancing the sense of vitality of thriving at work (Uygungil-Erdogan et al., 2025). Furthermore, digitization-oriented job demands allow employees to perform other high-value and creative tasks, further enhancing positive psychological experiences (AlNuaimi et al., 2022; P. Liu et al., 2024). Secondly, digitally knowledgeable employees can take control of their work and develop solutions, thus increasing employee job autonomy (Parker & Grote, 2022). This sense of control helps employees to adapt their work strategies to become more flexible (Y. Liu et al., 2024). In addition, digital technologies require employees to continuously learn (Hess et al., 2016; Vial, 2019), which can inspire them to investigate new fields and drive them to further their career development (P. Liu et al., 2024).

Furthermore, thriving at work, which provides powerful psychological energy resources for employees, could positively influence their motivation, resource access ability, and job crafting (Hobfoll, 2012). Job thriving endows employees with vitality and enhances their job resilience and adaptive functioning. This constitutes a psychological resource endowed to employees (Spreitzer et al., 2005). This helps employees cope with work stress and reduce work anxiety. Meanwhile, thriving employees are more intrinsically motivated to learn. They are more inclined to mobilize resources to learn and master digital work skills and knowledge (Chen et al., 2024), drive innovation in the workplace, and therefore enhance job-crafting behaviors (Riaz et al., 2018). In addition, thriving at work helps employees broaden their attention, thinking, and action, enabling them to engage in constructive job crafting activities (Yang et al., 2024). Overall, employees who are thriving at work have more psychological resources. They have greater motivation and ability to mobilize resources and access more resources and are more likely to engage in job-crafting behaviors. Based on this, the following hypotheses are proposed in this paper:

**H2a.**  *Thriving at work mediates the relationship between digitization-oriented job demands and job crafting*.

### 2.3. Resource Loss Pathways—The Mediating Role of Workplace Anxiety

Workplace anxiety, characterized by unease and tension in response to perceived threats, is a stress reaction commonly observed in organizational settings (McCarthy et al., 2016). It has been shown that task features and workplace anxiety are related (Y. Liu et al., 2024) in government agencies undergoing digital change (P. Liu et al., 2024). The increasing job demands for digital proficiency require employees to innovate and continuously learn new technologies (Chen et al., 2024). Employees may feel under pressure to adjust quickly and successfully (Verhoef et al., 2021), which could lead to negative feelings of worry and job insecurity (P. Liu et al., 2024). A recent study found that 51% of workers struggle to keep pace with their companies’ rapid adoption of digital technologies, resulting in heightened anxiety and other negative emotions (Blanchard, 2018).

According to COR theory, workplace stressors may result in negative emotions such as anxiety (Chen et al., 2024). The alterations of the working environment may precipitate further stress, thereby depleting their resources and intensifying their psychological distress (Aguiar-Quintana et al., 2021). Digitization-oriented job demands have undoubtedly transformed the traditional work nature and engendered heightened uncertainty (Chen et al., 2024). The civil servants’ job environments and content are becoming increasingly challenging and demanding (Parker & Grote, 2022), which may impose a psychological burden, depleting psychological resources and increasing workplace anxiety (Tang et al., 2023). Digitization-oriented job demands may increase employees’ workplace anxiety via the resource depletion pathway (Hobfoll et al., 2018). Meeting digitization-oriented job demands requires resources. This may be perceived as a burden for employees (Koo et al., 2021). Governments’ utilization of digital technologies expects civil servants to possess digital proficiency (Cetindamar Kozanoglu & Abedin, 2021). To meet the job demands related to digitization, workers must devote more time and effort to learn new and technology skills and knowledge (T.-J. Wu et al., 2024). Further, if employees have difficulty mastering technology skills and knowledge, it means that they lack the resources to recover from their efforts and may easily suffer from the anxiety associated with resources loss (Chen et al., 2024).

According to COR, individuals will activate their resource defense mechanisms when faced with resource loss (Hobfoll et al., 2003). Individuals minimize the loss of resources and conserve current resources by decreasing proactive behaviors such as withdrawal behaviors (Chen et al., 2024; Tang et al., 2023). Therefore, when digitization-oriented job demands create anxiety for employees, they may perceive resources as scarce and consequently reduce the investment of resources, which may result in work withdrawal behaviors increasing. Additionally, studies have shown anxiety at work makes workers avoid difficult situations and hinders their ability to absorb information (Y. Liu et al., 2024). This tendency increases employees’ reactive workplace behaviors (Du et al., 2023). In conclusion, this study put up the following theories:

**H2b.**  *Work anxiety mediates the relationship between digitization-oriented job demands and work withdrawal*.

### 2.4. The Moderating Role of the Regulatory Focus

Individuals adjust their cognitive processes and behaviors to adapt to changing environments, reduce the discrepancy between perceived reality and desired outcomes, and achieve their goals. This process is known as self-regulation (Peng et al., 2021). Individual self-regulatory approach or tendency is known as the regulatory focus, which is an important personality. Regulatory focus influences significant differences in how people react to external stimuli and reflects the human tendency to avoid injury and gain (Higgins, 1997; Lanaj et al., 2012). Regulatory focus theory classifies individuals’ motivational orientations into two distinct categories based on goal pursuit patterns: (1) prevention focus emphasizing safety and responsibility maintenance, and (2) promotion focus prioritizing advancement and aspiration fulfillment (Higgins, 1998).

Promotion-focused individuals demonstrate heightened sensitivity to developmental opportunities, and show a higher tendency for advancement, success, and development, pursuing success through proactive influence rather than failure avoidance (P. Liu et al., 2024). Conversely, prevention-focused individuals exhibit greater loss aversion, prioritizing risk mitigation through status quo preservation. They are worried about accountability, safety, and protection, and are more sensitive to unfavorable consequences (Brenninkmeijer et al., 2010). Consistent with the regulatory focus theory, individual regulatory focus is context-sensitive (Friedman & Förster, 2001; Higgins et al., 2001). Studies have shown that individuals with different focus demonstrate divergent environmental perceptions and psychological behavioral patterns (Johnson et al., 2015; Z. Liu & Brockner, 2015). Promotion-focused individuals are more sensitive to the positive features of the work environment, have better psychological experiences, and display more positive behaviors, while employees with prevention focus pay more attention to negative information and display more negative and passive behaviors (Lanaj et al., 2012). Consequently, when confronted with digitization-oriented job demands, individuals with different focus develop fundamentally different psychological states and behavioral outcomes.

In specific contexts and tasks, digital work demands can be considered a source of pressure and challenge (T.-J. Wu et al., 2025). Individuals will cope with stressful situations through resource accumulation and protection, and differences in resource acquisition strategies can be significantly influenced by an individual’s regulatory focus (Hobfoll et al., 2003; Lanaj et al., 2012). Promotion-focused individuals are more confident in dealing with digitization-oriented job demands and tend to view them as resource investment opportunities (Brenninkmeijer et al., 2010; Peng et al., 2021). Empirical studies have shown that promotion-focused individuals exhibit higher self-efficacy in digital tasks, are able to reframe work stress as a growth opportunity, and are more motivated and enthusiastic about learning new digital skills and knowledge. As a result, they thrive at work and their job-crafting behaviors increase (P. Liu et al., 2024). This difference is particularly significant in the public sector context. Research suggests that because public organizations often face institutional rigidity and resource constraints (Jung & Camarena, 2024), promotion-focused employees are more adept at using limited resources to create digital adaptation paths. Therefore, this study puts forth the following hypothesis:

**H3a.**  *Promotion focus moderates the relationship between digitization-oriented job demands and thriving at work. The positive effect of digitization-oriented job demands on thriving at work is more substantial when the promotion focus is higher*.

**H4a.**  *Promotion focus moderates the mediating effect of thriving at work in the positive relationship between digitization-oriented job demands and job crafting. The mediating effect of thriving at work between digitization-oriented job demands and job crafting is more substantial when the promotion focus is higher*.

In contrast, individuals with a prevention focus are more concerned with responsibility fulfillment and risk aversion (Mansour & Tremblay, 2020), and are more likely to perceive digital demands as a threat of resource depletion, which triggers resource defense behaviors. Specifically, when confronted with digitization-oriented job demands, they are more likely to perceive the situation as stressful and challenging, which may result in negative emotions such as fear, dread, and anxiety (P. Liu et al., 2024). Prevention-focused employees, when influenced by negative emotions such as anxiety, tend to either avoid measures or engage in negative behaviors such as work withdrawal (Peng et al., 2021). Individuals with a prevention-focused orientation are more concerned with the potential consequences of failure to meet demands and are particularly sensitive to adverse outcomes. This sensitivity can manifest as a lack of energy or reluctance to learn new digital technologies, which may lead to increased work withdrawal behaviors (Chen et al., 2024; Zhao & Liu, 2024). Research has shown that in the public sector, prevention-focused employees are overly concerned with “hindering needs” (e.g., the risk of technological failure). This leads to a reduction in motivation and ability to access technical knowledge resources, and ultimately, positive work behaviors are significantly reduced. Therefore, we propose the following hypothesis:

**H3b.**  *Prevention focus moderates the relationship between digitization-oriented job demands and workplace anxiety. The positive effect of digitization-oriented job demands on workplace anxiety is more substantial when the prevention focus is higher.*

**H4b.**  *Prevention focus moderates the mediating effect of workplace anxiety in the positive relationship between digitization-oriented job demands and work withdrawal. The mediating effect of workplace anxiety between digitization-oriented job demands and work withdrawal is more substantial when the prevention focus is higher*.

## 3. Methods

### 3.1. Sample and Collection

A questionnaire based on an online survey was distributed to fellow MPA students at universities in Southwest China to recruit civil servants interested in this study. These students then disseminated the questionnaire through snowball sampling, contacting colleagues interested in participating in this study.

This study used a three-wave data collection strategy with two-week gaps between waves to lessen the possible impact of common methodological bias. The mobile phone numbers of the participants were used to match the three sets of surveys. A total of 1122 questionnaires were received, with 873 deemed valid and representing a valid response rate of 77.81%. This figure excludes incomplete questionnaires, defined as those exhibiting a consistent response pattern and those not matching. The demographic variables, or control variables, are described by the specifications in Table 1.

### 3.2. Measures

Mature scales were used to measure all variables, and all English scale items were translated into Chinese utilizing a back-translation process in this study. A 5-point Likert scale was used for scoring.

A six-item scale was used to evaluate the digitization-oriented job demands (Janssen, 2000). The regulatory focus was measured using the scale developed by Wallace et al. (2009). There are 12 items on the scale, six representatives, and a focus on prevention and promotion. The workplace anxiety was assessed using an 8-item scale (McCarthy et al., 2016). The thriving at work was assessed using a 10-item scale (Porath et al., 2012), the work withdrawal was evaluated using a 12-item scale (Lehman & Simpson, 1992), and the job crafting was assessed using a 6-item scale (Leana et al., 2009). The internal consistency coefficients for all scales exceeded 0.7. This indicates that the selected scales exhibited good reliability. The titles of each scale are provided in Supplementary Materials.

### 3.3. Data Analysis

The data in this study were analyzed using Amos 24.0 and SPSS 27.0. SPSS 27.0 was used for descriptive statistical analysis, correlation analysis, and reliability testing. Amos 24.0 was used for hypothesis testing and validation factor analysis. Bias correction was applied using 2000 resamples and 95% CI to test and moderate mediation.

## 4. Results

### 4.1. Confirmatory Factor Analysis

Using a confirmatory factor analysis (CFA), the discriminant validity of the seven main components was evaluated. With χ^2^/df = 2.72, *p* < 0.001, CFI = 0.98, TLI = 0.97, RMSEA = 0.03, SRMR = 0.04, the results demonstrated that the suggested seven-factor model outperformed the other models (Table 2). Based on CFA, these results show that the proposed model is valid.

### 4.2. Common Method Bias

According to the findings of Harman’s one-way analysis of variance, the first principal component factor explained 37.68% of the variance, which was less than 40% (Podsakoff et al., 2003), and the unrotated exploratory factor analysis resolved seven factors, which accounted for 74.68% of the total variance explained. Furthermore, the fit superiority of the one-factor model is significantly lower than that of the seven-factor model, as Table 2 illustrates. In addition, the common method bias was further examined in this study by using the method of controlling unmeasurable latent factors. The results showed that there was no significant improvement in the comparison of model fit indices (Δ CFI = 0.03, Δ TLI = 0.02, Δ RMSEA = 0.005, Δ SRMR = 0.01) after adding method latent factors. The findings suggest that this study’s common technique bias is insignificant.

### 4.3. Reliability and Validity Analysis and Descriptive Statistics

The mean, standard deviation, and correlation between the research variables are displayed in Table 3. Regarding reliability, the Cronbach α of each scale was greater than 0.7, and the combined reliability CR values were higher than 0.7, which indicated that the sample had good reliability (Fornell & Larcker, 1981; Taber, 2018). Regarding validity, the factor loadings of the validated factor analysis were all greater than 0.6, and the average variance extracted (AVE) were all greater than 0.5, which indicated that the data in this study had good convergent validity (Fornell & Larcker, 1981).

### 4.4. Hypothesis Testing

In Amos 24.0, this study used structural equation modeling to evaluate hypotheses. As anticipated, job crafting and digitization-oriented job demands had a positive correlation (B = 0.41, *p* < 0.001), supporting hypothesis 1a (Table 4). Work withdrawal was positively correlated with digitization-oriented job demands (B = 0.30, *p* < 0.001), supporting hypothesis 1b. The current study employed the conditional indirect effects procedure (Preacher et al., 2010) to confirm the mediation effect of workplace anxiety and thriving at work. Hypothesis 2a was supported by the significant indirect influence of digitization-oriented job demands on job crafting through thriving at work (indirect effect = 0.07, 95% CI = [0.03, 0.15]). Hypothesis 2b was supported by the significant impact of digitization-oriented job demands on work withdrawal through workplace anxiety (indirect effect = 0.14, 95% CI = [0.10, 0.20]).

**Figure 2:**
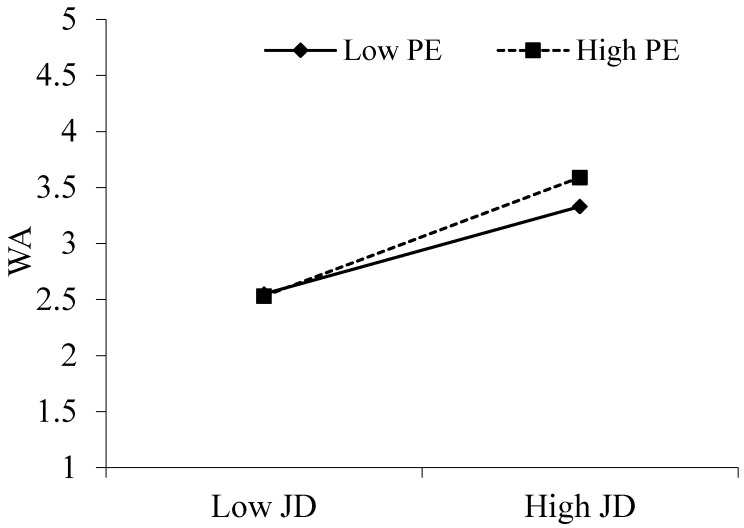
The moderation effect of PE on the relationship between JD and WA.

**Figure 3:**
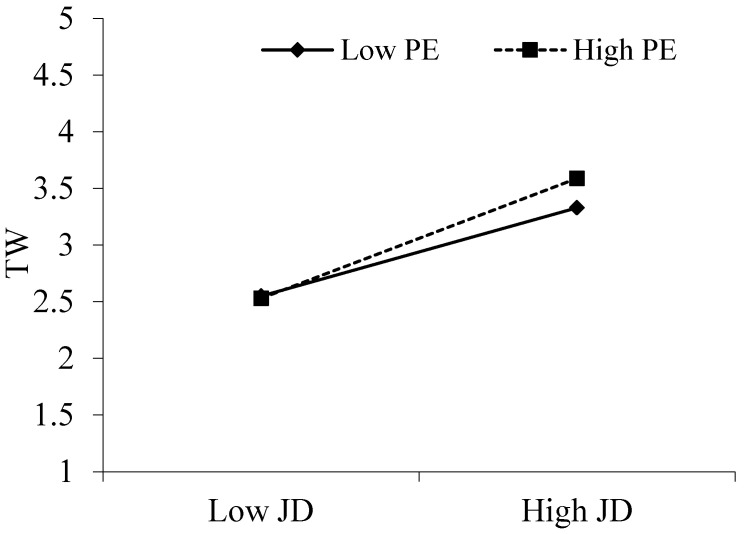
The moderation effect of PO on the relationship between JD and TW.

Table 4 shows the moderating effects of prevention and promotion focus. According to Table 4, the interaction term between digitization-oriented job demands and promotion focus positively correlated with thriving at work (B = 0.15, *p* < 0.001). Furthermore, Figure 2 shows that the positive correlation between digitization-oriented job demands and thriving at work became stronger at high promotion focus (B = 0.10, *p* < 0.001) compared to the positive correlation between digitization-oriented job demands and thriving at work at low promotion focus (B = 0.05, *p* < 0.05), which supports Hypothesis 3a. Furthermore, there was a significant moderating mediator index (B = 0.02, *p* < 0.05, 95% CI = [0.01, 0.04]). There was support for hypothesis 4a. Workplace anxiety was positively connected with the interplay between prevention focus and digitization-oriented job demands (B = 0.07, *p* < 0.05). Additionally, Figure 3 supports Hypothesis 3b by demonstrating that the positive association between digitization-oriented job demands and workplace anxiety increased at high prevention focus (B = 0.16, *p* < 0.001) in contrast to the positive correlation at low prevention focus (B = 0.12, *p* < 0.001). Furthermore, there was a significant moderator-mediator index (B = 0.02, *p* < 0.001, 95% CI = [0.00, 0.04]). There was support for hypothesis 4b (Table 5).

## 5. Discussion

### 5.1. Theoretical Contributions

The present study contributes to existing theoretical frameworks in the following ways.

First, this paper investigates how job demands affect employee behavior based on a digital perspective, extending related research. In digitization content, the impact of job demands changes related to the employee behavior has not been adequately studied. This study adds to the body of knowledge already available on the subject.

Second, as civil servants play a key role in achieving organizational digital transformation and performing public service functions (Jung & Camarena, 2024), it is imperative to pay attention to their cognitive and behavioral responses to digitization-oriented job demands. However, current research on the impact of digital technology innovations in the public sector, the scholars have mainly focused on its organizational level (e.g., departmental performance (Chatterjee et al., 2023), management processes (Hess et al., 2016), and decision-making efficiency (Vial, 2019), while ignoring the human factor, to which this study contributes.

Third, previous studies on the impact of the organizational application of digitization on employees have produced conflicting or ambiguous results (Chen et al., 2024; P. Liu et al., 2024). This study verifies that digitization-oriented job demands can have a double-edged effect on employees, integrating the insights of previous studies. Further, this study provides a more comprehensive framework for understanding employee psychology and behavior in the process of digitization in the public sector.

Finally, our study explains the double-edged sword effect of digitization-oriented job demands from the perspective of resource loss and enrichment, and further validates the COR theory (Hobfoll, 2012). Furthermore, our study illustrates that regulatory focus significantly influences employee work behavior and outcomes, aligning with existing perspectives (P. Liu et al., 2024) and reinforcing the tenets of the regulatory focus theory.

### 5.2. Practical Implications

Our research is a contribution to how organizational managers can develop management strategies for the digital labor demands in the digital era. And our research also helps civil servants to better adapt their psychology and behavior in the face of organizational digital transformation.

First, our study demonstrates that digitization-oriented job demands have a dual impact on employees. As some employees experience difficulty in adapting to organizational digital changes (Khan et al., 2024; T.-J. Wu et al., 2025), which can result in technology anxiety (Jung & Camarena, 2024), job insecurity (Lemmer et al., 2023), and other adverse reactions. Therefore, public sector managers should pay attention to the comprehensive impact of digitization-oriented job demands (Jung & Camarena, 2024) and put forward targeted demands based on job characteristics, employee traits, and so on to maximize the benefits of digitization.

Second, public sector managers can enhance employees’ sense of thriving and reduce anxiety by providing requisite resources (Jung & Camarena, 2024). For instance, comprehensive digital skills training (Feroz et al., 2023), the provision of requisite hardware support, attention paid to employees’ negative emotions, and enhancing employees’ competence in digitization-oriented job demands. As for civil servants themselves, our research contributes to helping civil servants better adapt their perceptions and behaviors in the digital context.

Furthermore, our study revealed that employees’ regulatory focus significantly determines their response to digitization-oriented job demands. Managers should be adept at identifying and screening promotion-focused employees (Sacramento et al., 2013) and placing them in positions that require high digital competence. At the same time, managers should also consider strategies to increase the promotion focus of those employees who focus on prevention.

It is also noteworthy that employee relationships are influenced by work stress (Ghi et al., 2022). Public sector managers should foster a knowledge-sharing culture, encouraging those employees who have high digital competence to help their colleagues. It is also noteworthy that younger employees display more positive emotions and behaviors when confronted with digitization-oriented job demands than their older counterparts. Consequently, it would be prudent to structure work that aligns with employees’ age characteristics.

### 5.3. Limitations and Future Research

It is recommended that future research addresses the following limitations of the present study. First, employee self-reports were used to determine every variable in this study. Harman’s one-factor test might not have adequately solved this problem despite being used to combat CMV. Future research would benefit from using more scientific measures to improve the data’s robustness. Second, influencing employees’ psychological and work-related behaviors in the digital workplace requires the use of a variety of psychological mechanisms. The current study examined how workplace anxiety and prosperity impacted employees’ job crafting and withdrawal behaviors. Additional work practices and other ways job expectations impact employees in the digital world could be the subject of future research. In summary, the current study did not look at other possible moderators and instead focused on the moderating effect of defensive/facilitative moderating focus at the person level. Future studies might benefit from looking at the moderating effects of team-level (such as helpful behaviors) and organizational-level (such as leader support) factors on the connection between employee behaviors and digitization-oriented job expectations. In addition, this study relied on snowball sampling of MPA students to obtain a sample, which may not be representative of Chinese public sector employees. Although our study initially sent the questionnaire to a dozen or so MPA students working at the grassroots level and asked them to send it to colleagues who might be interested in this study, this is still not representative of the broader population of civil servants in China, and could be investigated by going deeper into the public sector that it characterizes in the future. Finally, this study was conducted with Chinese public sector employees at the grassroots level, but we have not yet conducted a specific segmentation for different functions/types to explore their behavior in the context of digital work. In the future, more specific public sectors (e.g., education, customs, etc.) could be investigated.

## Figures and Tables

**Table 1:** Descriptive statistics of demographic variables.

Variable	Categories	Code	Frequency	Percentage
Sex	Male	1	456	52.2
	Female	0	417	47.8
Education	College and below	1	47	5.4
	Bachelor’s Degree	2	262	30.0
	Master’s degree	3	564	64.6
Rank	Section Chief	1	368	42.2
	Deputy Section	2	291	33.3
	Full Section	3	214	24.5

**Table 2:** Results of confirmatory factor analyses.

Model	Factors	χ^2^	df	χ^2^/df	CFI	TLI	RMSEA	SRMR
7-factor model	JD; PO; PE; TW; WA; JC; WD	2451.70	901	2.72	0.98	0.97	0.03	0.04
6-factor model	JD + PO; PE; TW; WA; JC; WD	11,642.85	1112	10.47	0.73	0.71	0.10	0.11
5-factor model	JD + PO + PE; TW; WA; JC; WD	15,991.17	1117	14.32	0.69	0.68	0.12	0.13
4-factor model	JD + PO + PE + TW; WA; JC; WD	23,544.73	1121	21.00	0.55	0.52	0.13	0.14
3-factor model	JD + PO + PE + TW + WA; JC; WD	26,717.26	1124	23.77	0.52	0.49	0.14	0.16
2-factor model	JD + PO + PE + TW + WA + JC; WD	29,345.35	1126	26.06	0.41	0.39	0.16	0.17
1-factor model	JD + PO + PE + TW + WA + JC + WD	31,257.59	1127	27.74	0.41	0.38	0.17	0.19

Note. JD = digitization-oriented job demands; PO = promotion focus, PE = prevention focus; TW = thriving at work; WA = workplace anxiety; JC = job crafting; WD = work withdrawal.

**Table 3:** Means, standard deviations, and correlations of variables.

	1	2	3	4	5	6	7	8	9	10	11
1. Gender (T1)	-										
2. Age (T1)	0.07 *	-									
3. Education (T1)	0.05	0.14 **	-								
4. Rank (T1)	−0.08 *	−0.16 **	−0.05	-							
5. JD (T1)	−0.11 **	0.01	0.04	0.04	**0.76**						
6. PO (T1)	−0.16 **	0.09 **	−0.01	0.06	0.35 **	**0.86**					
7. PE (T1)	−0.23 **	0.09 **	−0.03	0.04	0.21 **	0.36 **	**0.80**				
8. TW (T2)	−0.10 **	0.06	−0.01	0.02	0.48 **	0.33 **	−0.22 **	**0.89**			
9. WA (T2)	−0.12 **	0.06	−0.01	0.03	0.49 **	−0.37 **	0.23 **	−0.48 **	**0.89**		
10. JC (T3)	−0.10 **	0.02	−0.01	−0.03	0.44 **	0.11 **	−0.14 **	0.33 **	−0.35 **	**0.82**	
11. WD (T3)	−0.17 **	0.04	−0.04	0.04	0.56 **	0.40 **	0.27 **	−0.52 **	0.57 **	−0.62 **	**0.73**
Mean	0.48	40.53	2.59	1.82	3.83	2.57	2.55	3.64	3.64	3.73	3.83
PE	0.50	9.06	0.59	0.80	0.63	0.99	1.01	0.67	0.61	0.70	0.51
Cronbach’s α	-	-	-	-	0.95	0.96	0.96	0.98	0.92	0.92	0.89
CR	-	-	-	-	0.81	0.98	0.93	0.97	0.96	0.96	0.90
AVE	-	-	-	-	0.58	0.74	0.64	0.80	0.80	0.67	0.53

Note. *n* = 873; Bolding indicates the square root of AVE; T1 = Time1; T2 = Time2, T3 = Time3 * p < 0.05; ** *p* < 0.01.

**Table 4:** Summary of path-analytic results.

Variables	TW		JC		WA		WD	
	B	SE	B	SE	B	SE	B	SE
JD	0.46 ***	0.03	0.41 ***	0.04	0.46 ***	0.03	0.30 ***	0.02
PO	0.08 ***	0.02						
JD × PO	0.15 ***	0.03						
PE					0.06 **	0.02		
JD × PE					0.07 **	0.03		
TW			0.16 ***	0.04				
WA							0.30 ***	0.03
Gender	−0.05	0.04	−0.08	0.04	−0.05	0.04	−0.09 **	0.03
Age	0.00	0.00	0.00	0.00	0.00	0.00	0.00	0.00
Education	−0.03	0.04	−0.03	0.04	−0.03	0.03	−0.05 **	0.02
Type	−0.02	0.03	−0.05	0.03	0.00	0.02	0.01	0.02

Note. B = path coefficients; SE = standardized errors; path coefficients are standardized; model fit statistics: χ^2^/df = 1.152; CFI = 0.971; TLI = 0.970; RMSEA = 0.023; SRMR = 0.041; ** p < 0.01; *** *p <* 0.001.

**Table 5:** Bootstrapping results for testing mediation effect and moderated mediation effect.

**Moderator: PO**	**JD→TW→JC**		
	**B**	**SE**	**95% Boot CI**
Indirect effect	0.07 **	0.02	[0.03, 0.15]
Direct effect	0.41 ***	0.04	[0.33, 0.48]
High (+PE)	0.10 ***	0.03	[0.04, 0.15]
Low (−PE)	0.05 **	0.02	[0.02, 0.09]
Index	0.02 **	0.01	[0.01, 0.04]
**Moderator: PE**	**JD→WA→WD**		
	**B**	**SE**	**95% Boot CI**
Indirect effect	0.14 ***	0.02	[0.10, 0.20]
Direct effect	0.30 ***	0.02	[0.25, 0.34]
High (+PE)	0.16 ***	0.02	[0.11, 0.21]
Low (−PE)	0.12 ***	0.02	[0.08, 0.16]
Index	0.02 **	0.01	[0.00, 0.04]

Note. B path coefficients; SE = standardized errors; CI that excludes zero indicates that the indirect effects are significant; path coefficients are standardized; number of bootstrap samples is 2000; level of confidence is 95%; low refers to one standard deviation below the mean of the moderator; high refers to one standard deviation above the mean of the moderator; ** *p* < 0.01; *** *p* < 0.001.

## Data Availability

The data that support the findings of this study are available on request from the corresponding author. The data are not publicly available due to privacy or ethical restrictions.

## Supplementary Materials

The following supporting information can be downloaded at: https://www.mdpi.com/article/10.3390/bs15030378/s1.

## References

[B1-behavsci-15-00378] Aguiar-Quintana T., Nguyen T. H. H., Araujo-Cabrera Y., Sanabria-Díaz J. M. (2021). Do job insecurity, anxiety and depression caused by the COVID-19 pandemic influence hotel employees’ self-rated task performance? The moderating role of employee resilience. International Journal of Hospitality Management.

[B2-behavsci-15-00378] AlNuaimi B. K., Kumar Singh S., Ren S., Budhwar P., Vorobyev D. (2022). Mastering digital transformation: The nexus between leadership, agility, and digital strategy. Journal of Business Research.

[B3-behavsci-15-00378] Bakker A. B., de Vries J. D. (2021). Job demands-resources theory and self-regulation: New explanations and remedies for job burnout. Anxiety Stress and Coping.

[B4-behavsci-15-00378] Blanchard O. (2018). On the future of macroeconomic models. Oxford Review of Economic Policy.

[B5-behavsci-15-00378] Brenninkmeijer V., Demerouti E., Le Blanc P. M., Hetty Van Emmerik I. J. (2010). Regulatory focus at work: The moderating role of regulatory focus in the job demands-resources model. Career Development International.

[B6-behavsci-15-00378] Cetindamar Kozanoglu D., Abedin B. (2021). Understanding the role of employees in digital transformation: Conceptualization of digital literacy of employees as a multi-dimensional organizational affordance. Journal of Enterprise Information Management.

[B7-behavsci-15-00378] Chatterjee S., Chaudhuri R., Vrontis D., Giovando G. (2023). Digital workplace and organization performance: Moderating role of digital leadership capability. Journal of Innovation & Knowledge.

[B8-behavsci-15-00378] Chen N., Zhao X., Wang L. (2024). The effect of job skill demands under artificial intelligence embeddedness on employees’ job performance: A moderated double-edged sword model. Behavioral Sciences.

[B9-behavsci-15-00378] Demerouti E., Bakker A. B. (2023). Job demands-resources theory in times of crises: New propositions. Organizational Psychology Review.

[B10-behavsci-15-00378] Demerouti E., Bakker A. B., Gevers J. M. P. (2015). Job crafting and extra-role behavior: The role of work engagement and flourishing. Journal of Vocational Behavior.

[B11-behavsci-15-00378] Du Y., Li J., Xu Q. (2023). Are you satisfied when your job fits? The perspective of career management. Baltic Journal of Management.

[B12-behavsci-15-00378] Elliott E. S., Dweck C. S. (1988). Goals: An approach to motivation and achievement. Journal of Personality and Social Psychology.

[B13-behavsci-15-00378] Feroz A. K., Zo H., Eom J., Chiravuri A. (2023). Identifying organizations’ dynamic capabilities for sustainable digital transformation: A mixed methods study. Technology in Society.

[B14-behavsci-15-00378] Fornell C., Larcker D. F. (1981). Evaluating structural equation models with unobservable variables and measurement error. Journal of Marketing Research.

[B15-behavsci-15-00378] Friedman R. S., Förster J. (2001). The effects of promotion and prevention cues on creativity. Journal of Personality and Social Psychology.

[B16-behavsci-15-00378] Ghi T., Thu N., Huan N., Trung N. (2022). Human capital, digital transformation, and firm performance of startups in Vietnam. Management.

[B17-behavsci-15-00378] Hess T., Matt C., Benlian A., Wiesbck F. (2016). Options for formulating a digital transformation strategy. MIS Quarterly Executive.

[B18-behavsci-15-00378] Higgins E. T. (1997). Beyond pleasure and pain. American Psychologist.

[B19-behavsci-15-00378] Higgins E. T. (1998). Promotion and prevention: Regulatory focus as a motivational principle. Advances in Experimental Social Psychology.

[B20-behavsci-15-00378] Higgins E. T., Friedman R. S., Harlow R. E., Idson L. C., Ayduk O. N., Taylor A. (2001). Achievement orientations from subjective histories of success: Promotion pride versus prevention pride. European Journal of Social Psychology.

[B21-behavsci-15-00378] Hill N. S., Axtell C., Raghuram S., Nurmi N. (2024). Unpacking virtual work’s dual effects on employee well-being: An integrative review and future research agenda. Journal of Management.

[B22-behavsci-15-00378] Hobfoll S. E. (2012). Conservation of resources and disaster in cultural context: The caravans and passageways for resources. Psychiatry: Interpersonal and Biological Processes.

[B23-behavsci-15-00378] Hobfoll S. E., Halbesleben J., Neveu J.-P., Westman M. (2018). Conservation of resources in the organizational context: The reality of resources and their consequences. Annual Review of Organizational Psychology and Organizational Behavior.

[B24-behavsci-15-00378] Hobfoll S. E., Johnson R. J., Ennis N., Jackson A. P. (2003). Resource loss, resource gain, and emotional outcomes among inner city women. Journal of Personality and Social Psychology.

[B25-behavsci-15-00378] Hou Y., Fan L. (2024). Working with AI: The effect of job stress on hotel employees’ work engagement. Behavioral Sciences.

[B26-behavsci-15-00378] Ingusci E., Signore F., Giancespro M. L., Manuti A., Molino M., Russo V., Zito M., Cortese C. G. (2021). Workload, techno overload, and behavioral stress during COVID-19 emergency: The role of job crafting in remote workers. Frontiers in Psychology.

[B27-behavsci-15-00378] Janssen O. (2000). Job demands, perceptions of effort-reward fairness and innovative work behaviour. Journal of Occupational and Organizational Psychology.

[B28-behavsci-15-00378] Johnson P. D., Smith M. B., Wallace J. C., Hill A. D., Baron R. A. (2015). A review of multilevel regulatory focus in organizations. Journal of Management.

[B29-behavsci-15-00378] Jung H., Camarena L. (2024). Street-level bureaucrats & AI interactions in public organizations: An identity based framework. Public Performance & Management Review.

[B30-behavsci-15-00378] Kaltiainen J., Hakanen J. J. (2024). Why increase in telework may have affected employee well-being during the COVID-19 pandemic? The role of work and non-work life domains. Current Psychology.

[B31-behavsci-15-00378] Khan A. N., Shahzad K., Khan N. A. (2024). Fostering accountability through digital transformation: Leadership’s role in enhancing techno-work engagement in public sector. Public Money & Management.

[B32-behavsci-15-00378] Koo B., Curtis C., Ryan B. (2021). Examining the impact of artificial intelligence on hotel employees through job insecurity perspectives. International Journal of Hospitality Management.

[B33-behavsci-15-00378] Lanaj K., Chang C.-H., Johnson R. E. (2012). Regulatory focus and work-related outcomes: A review and meta-analysis. Psychological Bulletin.

[B34-behavsci-15-00378] Leana C., Appelbaum E., Shevchuk I. (2009). Work process and quality of care in early childhood education: The role of job crafting. Academy of Management Journal.

[B35-behavsci-15-00378] Lehman W. E., Simpson D. D. (1992). Employee substance use and on-the-job behaviors. Journal of Applied Psychology.

[B36-behavsci-15-00378] Lemmer K., Jahn K., Chen A., Niehaves B. (2023). One tool to rule?—A field experimental longitudinal study on the costs and benefits of mobile device usage in public agencies. Government Information Quarterly.

[B37-behavsci-15-00378] Li J., Yang H., Weng Q., Zhu L. (2023). How different forms of job crafting relate to job satisfaction: The role of person-job fit and age. Current Psychology.

[B38-behavsci-15-00378] Liu P., Zhang F., Liu Y., Liu S., Huo C. (2024). Enabling or burdening?-the double-edged sword impact of digital transformation on employee resilience. Computers in Human Behavior.

[B39-behavsci-15-00378] Liu Y., Li Y., Song K., Chu F. (2024). The two faces of artificial intelligence (AI): Analyzing how AI usage shapes employee behaviors in the hospitality industry. International Journal of Hospitality Management.

[B40-behavsci-15-00378] Liu Z., Brockner J. (2015). The interactive effect of positive inequity and regulatory focus on work performance. Journal of Experimental Social Psychology.

[B41-behavsci-15-00378] Mansour S., Tremblay D.-G. (2020). How can organizations foster job crafting behaviors and thriving at work?. Journal of Management & Organization.

[B42-behavsci-15-00378] McCarthy J. M., Trougakos J. P., Cheng B. H. (2016). Are anxious workers less productive workers? It depends on the quality of social exchange. Journal of Applied Psychology.

[B43-behavsci-15-00378] Muneer S., Singh A., Choudhary M. H., Alshammari A. S. (2024). The mediating role of psychological empowerment on the relationship between digital transformation, innovative work behavior, and organizational financial performance. Behavioral Sciences.

[B44-behavsci-15-00378] Nix G. A., Ryan R. M., Manly J. B., Deci E. L. (1999). Revitalization through self-regulation: The effects of autonomous and controlled motivation on happiness and vitality. Journal of Experimental Social Psychology.

[B45-behavsci-15-00378] Parker S. K., Grote G. (2022). Automation, algorithms, and beyond: Why work design matters more than ever in a digital world. Applied Psychology.

[B46-behavsci-15-00378] Pei X., Guo J., Wu T.-J. (2025). How ambivalence toward digital–AI transformation affects taking-charge behavior: A threat–rigidity theoretical perspective. Behavioral Sciences.

[B47-behavsci-15-00378] Peng J., Cao F., Zhang Y., Cao Y., Zhang Y., Zhu X., Miao D. (2021). Reflections on motivation: How regulatory focus influences self-framing and risky decision making. Current Psychology.

[B48-behavsci-15-00378] Podsakoff P. M., MacKenzie S. B., Lee J.-Y., Podsakoff N. P. (2003). Common method biases in behavioral research: A critical review of the literature and recommended remedies. Journal of Applied Psychology.

[B49-behavsci-15-00378] Porath C., Spreitzer G., Gibson C., Garnett F. G. (2012). Thriving at work: Toward its measurement, construct validation, and theoretical refinement. Journal of Organizational Behavior.

[B50-behavsci-15-00378] Preacher K. J., Zyphur M. J., Zhang Z. (2010). A general multilevel SEM framework for assessing multilevel mediation. Psychological Methods.

[B51-behavsci-15-00378] Ramakrishnan S., Wong M. S., Chit M. M., Mutum D. S. (2024). The mediating role of occupational stress: A missing link between organisational intelligence traits and digital government service quality. International Journal of Quality & Reliability Management.

[B52-behavsci-15-00378] Riaz S., Xu Y., Hussain S. (2018). Understanding employee innovative behavior and thriving at work: A Chinese perspective. Administrative Sciences.

[B53-behavsci-15-00378] Rosse J. G., Hulin C. L. (1985). Adaptation to work: An analysis of employee health, withdrawal, and change. Organizational Behavior and Human Decision Processes.

[B54-behavsci-15-00378] Sacramento C. A., Fay D., West M. A. (2013). Workplace duties or opportunities? Challenge stressors, regulatory focus, and creativity. Organizational Behavior and Human Decision Processes.

[B55-behavsci-15-00378] Sandoval-Reyes J., Acosta-Prado J. C., Sanchís-Pedregosa C. (2019). Relationship amongst technology use, work overload, and psychological detachment from work. International Journal of Environmental Research and Public Health.

[B56-behavsci-15-00378] Spreitzer G., Sutcliffe K., Dutton J., Sonenshein S., Grant A. M. (2005). A socially embedded model of thriving at work. Organization Science.

[B57-behavsci-15-00378] Taber K. S. (2018). The use of Cronbach’s alpha when developing and reporting research instruments in science education. Research in Science Education.

[B58-behavsci-15-00378] Tang P. M., Koopman J., Mai K. M., De Cremer D., Zhang J. H., Reynders P., Ng C. T. S., Chen I.-H. (2023). No person is an island: Unpacking the work and after-work consequences of interacting with artificial intelligence. Journal of Applied Psychology.

[B59-behavsci-15-00378] Uygungil-Erdogan S., Şahin Y., Sökmen-Alaca A. İ., Oktaysoy O., Altıntaş M., Topçuoğlu V. (2025). Assessing the effect of artificial intelligence anxiety on turnover intention: The mediating role of quiet quitting in turkish small and medium enterprises. Behavioral Sciences.

[B60-behavsci-15-00378] Verhoef P. C., Broekhuizen T., Bart Y., Bhattacharya A., Qi Dong J., Fabian N., Haenlein M. (2021). Digital transformation: A multidisciplinary reflection and research agenda. Journal of Business Research.

[B61-behavsci-15-00378] Vial G. (2019). Understanding digital transformation: A review and a research agenda. The Journal of Strategic Information Systems.

[B62-behavsci-15-00378] Wallace J. C., Johnson P. D., Frazier M. L. (2009). An examination of the factorial, construct, and predictive validity and utility of the regulatory focus at work scale. Journal of Organizational Behavior.

[B63-behavsci-15-00378] Wu J., Gong X., Liu Y. (2022). Research on the influence mechanism of employees’ innovation behavior in the context of digital transformation. Frontiers in Psychology.

[B64-behavsci-15-00378] Wu T.-J., Liang Y., Wang Y. (2024). The buffering role of workplace mindfulness: How job insecurity of human-artificial intelligence collaboration impacts employees’ work–life-related outcomes. Journal of Business and Psychology.

[B65-behavsci-15-00378] Wu T.-J., Zhang R.-X., Zhang Z. (2025). Navigating the human-artificial intelligence collaboration landscape: Impact on quality of work life and work engagement. Journal of Hospitality and Tourism Management.

[B66-behavsci-15-00378] Yang Y., Wang X., Bell C. M. (2024). From thriving to task focus: The role of needs-supplies fit and task complexity. Current Psychology.

[B67-behavsci-15-00378] Zhang J., Su D., Smith A. P., Yang L. (2023). Reducing work withdrawal behaviors when faced with work obstacles: A three-way interaction model. Behavioral Sciences.

[B68-behavsci-15-00378] Zhao S., Liu Y. (2024). Job demands-resources on digital gig platforms and counterproductive work behavior. Frontiers in Psychology.

