# Supplementary material for: Master or Escape: Digitization-Oriented Job Demands and Crafting and Withdrawal of Chinese Public Sector Employees
Source: Behav Sci (Basel). 2025 Mar 17;15(3):378. doi: 10.3390/bs15030378 (PMC11939424; doi:10.3390/bs15030378)
Supplement: Supplementary file 1 [file behavsci-15-00378-s001.zip › behavsci-3474965-supplementary.pdf]

Supplementary Materials

Digitization-oriented job demands (Janssen, 2000)

1. I must work in a timely and efficient manner to facilitate my organization's adaptation to the digital age.
2. The volume of work required due to digital changes is considerable.
3. The effort required to complete tasks related to digital-oriented changes is approximately double that which would otherwise be necessary.
4. I am required to complete digitization-related tasks within a minimal timeframe.
5. I consider the working environment to be less than ideal for meeting the demands of digitalization.
6. I am responsible for dealing with a large backlog of work related to digital change.

Thriving at work (C. Porath et al., 2012)

1. At work, I find myself learning often.
2. I continue to learn more at work as time goes by.
3. At work, I see myself continually improving.
4. At work, I am learning.
5. At work, I have developed a lot as a person.
6. At work, I feel alive and vital.
7. At work, I have energy and spirit.
8. At work, I do feel very energetic.
9. At work, I feel alert and awake.
10. At work, I am looking forward to each new day.

Workplace anxiety (McCarthy et al., 2016)

1. I am overwhelmed by thoughts of doing poorly at work.
2. I worry that my work performance will be lower than others.
3. I feel nervous and apprehensive about being unable to meet performance targets.
4. I worry about not receiving a positive job performance evaluation.
5. I often feel anxious that I cannot perform my job duties in the time allotted.
6. I worry about whether others consider me to be a good employee for the job
7. I worry that I will not be able to manage the demands of my job successfully.
8. Even when I try as hard as possible, I still worry about whether my job performance will be good enough.

Work withdrawal (Lehman & Simpson, 1992)

1. I have absenteeism at work.
2. I will talk about non-work topics with colleagues at work.
3. I have left work for unnecessary reasons.

4. I daydream at work.
5. I spend work time on personal matters.
6. I put less effort into my work than I should.
7. I have thoughts of leaving my job.
8. I want someone else to do my job.
9. I have left work early without permission.
10. I have taken lunch or breaks longer than allowed.
11. I have taken supplies or equipment without permission.
12. I have fallen asleep on the job.

Job crafting (Leana et al., 2009).

1. I will take it upon myself to introduce new methods and improve work procedures.
2. I will change work procedures that I consider to be unproductive and of secondary importance
3. I will seek to change my working methods to make myself more relaxed.
4. I tend to rearrange the equipment in my work area by myself.
5. I will organize special events at work (e.g., birthday celebrations for colleagues)
6. I will bring my additional materials from home for the job.

Promotion focus (Wallace & Chen, 2006)

1. I get many things done at work
2. I get my work done no matter what
3. I can get much work done in a short period
4. I am passionate about work activities that make me successful
5. I strive to achieve work accomplishments
6. I recognize how many tasks I can complete

Prevention focus (Wallace & Chen, 2006)

1. I comply with rules and regulations as much as possible.
2. I prefer to do my work tasks correctly.
3. I try to fulfill my obligations at work to the best of my ability.
4. I pay attention to my job responsibilities.
5. I try to fulfill my work obligations.
6. I care about the details of my work.
